# An original approach to measure ligand/receptor binding affinity in non-purified samples

**DOI:** 10.1038/s41598-022-09217-6

**Published:** 2022-03-30

**Authors:** Estelle Rascol, Anouk Dufourquet, Rim Baccouch, Pierre Soule, Isabel D. Alves

**Affiliations:** 1 Univ. Bordeaux, CNRS, Bordeaux INP, CBMN, UMR 5248, 33600 Pessac, France; 2 Application Team, NanoTemper Technologies GmbH, Munich, Germany

**Keywords:** Membrane biophysics, Biochemistry, Biophysics

## Abstract

Several biochemical and biophysical methods are available to determine ligand binding affinities between a biological target and its ligands, most of which require purification, labelling or surface immobilisation. These measurements, however, remain challenging in regards to membrane proteins, as purification processes require their extraction from their native lipid environment, which may in turn impact receptor conformation and functionality. In this study, we have developed a novel experimental procedure using microscale thermophoresis (MST) directly from cell membrane fragments, to determine different ligand binding affinities to a membrane protein, the dopamine D2 receptor (D2R). In order to achieve this, two main challenges had to be overcome: determining the concentration of dopamine D2R in the crude sample; finding ways to minimize or account for non-specific binding of the ligand to cell fragments. Using MST, we were able to determine the D2R concentration in cell membrane fragments to approximately 36.8 ± 2.6 pmol/mg. Next, the doses-responses curves allowed for the determination of K_D_, to approximately 5.3 ± 1.7 nM, which is very close to the reported value. Important details of the experimental procedure have been detailed in this paper to allow the application of this novel method to various membrane proteins.

## Introduction

G protein-coupled receptors (GPCRs) constitute a large family of integral membrane proteins. As they can induce very different intracellular signalling events upon activation by extracellular stimuli (small molecules, peptides, light, odorants, etc.), they represent one of the most important drug target families. Nevertheless, it is still challenging to assess their pharmacological properties in their physiological environment. Defining ligand binding affinity is one of the most important properties, in particular for the development of new therapeutic agents. Among the biochemical or biophysical approaches available to determine ligand binding affinity, some strategies require labelling (in-solution approaches) and others the capturing (sensor-based technology approaches) of one of the partners. To do so, there are two general strategies: (1) performing measurements directly on cells or cell fragments; (2) extracting the receptor from its natural environment, isolating, purifying and quantifying it before measuring its ligand affinity^1^. While the second approach allows for certain parameters to be better controlled (for instance the lipid and protein-membrane composition), the first strategy is more commonly employed due to its simplicity and the fact that receptor isolation and purification can alter the native protein functionality^2^. To illustrate, purification frequently requires the addition of tags to the native protein, thus modifying the protein sequence. Additionally, the solubilisation of membrane proteins from cell membranes may also affect protein functionality^3^. Furthermore, recent findings have shown the role of membrane components, such as specific lipids, in protein functionality in particular involving GPCRs^4^.

It is therefore important to develop methods and protocols to characterize GPCRs in their native environment. One of the pioneer methods, and still of great use nowadays is the radiolabel assay. While being extremely sensitive (K_D_ can be determined in the picomolar range)^5^, there are many drawbacks of this assay, including the necessity to have specific laboratory equipment, dedicated and isolated laboratory space and trained personnel to safely deal with the hurdle of using radioactivity^6^. To avoid such problems, within the last two decades or so, several approaches based on fluorescence measurements have been reported^7^, often associated with the development of new methods and the establishment of protocols. The most well-known is based on the fluorophore rotational speed measurement, such as fluorescence polarization (FP), or on resonance energy transfer, such as homogeneous time-resolved fluorescence (HTRF), both of which exploiting light properties.

Microscale thermophoresis (MST) has been developed as a low sample consumption and versatile method for the quantification of various protein interactions, including ligand/receptor interplay. The method relies on the observation of fluorescence variation upon a sudden temperature modification of a few degrees, induced by an infrared laser. This temperature change induces two interconnected phenomena^8^. On one hand, the temperature gradient induces diffusion of molecules, relating to thermophoresis. On the other hand, increasing thermal agitation leads to the dimming of the dye, specifying the temperature-related intensity change (TRIC). The combination of the two phenomena together describe MST, with the change in intensity observed upon temperature modification being linked to the size, the charge and the hydration of the labelled molecule, referred to as the target. Any modification upon ligand binding to the target will ultimately result in a modification of the MST signal, thus making the technique sensitive to even minute changes, such as ion binding to a labelled protein. Last, but not least, since the technique relies on fluorescence emission, it is quite sensitive and uses only a few nM of the target and only 10 µL sample volumes sample volumes. In order to achieve this, however, one of the two partners of interest, the ligand or the target, needs to be fluorescent. Purified proteins may be followed thanks to tryptophan fluorescence^9^ or with labelling via lysine or cysteine residues^10^. MST is also sensitive enough to work in complex matrices such as blood samples or cell lysates. Such approaches usually require the target to be specifically labelled, for example with a fused GFP protein, which can be used to investigate protein–protein interactions^11^, or ligand/target binding affinity^12^. Tagged proteins (Histidine Tag, SNAP tag) can also be specifically coupled to a fluorophore using commercially available kits^13^, or alternatively fluorescently labelled ligands can be used^14^.

Several challenges appear when working with very complex matrices, in particular with the determination of the concentration of the biomolecule of interest as well as with non-specific binding to matrix components. While, MST has been applied to the study of soluble proteins in complex matrices and in non-purified forms, it hasn’t yet been the case for membrane proteins (including GPCRs), where inherent additional challenges exist. To the best of our knowledge, all MST studies involving GPCRs have been performed using purified systems^9,15–17^. In this paper, we propose a method to characterize ligand binding to a model GPCR, the dopamine D2R receptor (D2R), using MST with minimal manipulation of the crude protein, following expression in mammalian cell plasma membrane.

## Results

### Determination of D2R concentration in cell membrane fragments

To study the ligand/receptor interaction, aim of our present work, the ligand spiperone—Cy5 (a known D2R antagonist)^18^ and the D2R consisting of non-purified membrane fragments (HEK cells expressing the D2R, HekD2) were used. The ligand, being the fluorescent partner in this study, was maintained at a fixed concentration, while the D2R concentration was varied. The concentration of the D2R in the membrane sample is unknown, as a result, the first goal was to determine the D2R concentration using MST. To do so, a titration experiment was performed, with a fixed spiperone—Cy5 concentration above the K_D_ value and a varying D2R concentration. Taking into account that a 1/1 stoichiometry exists in the D2R/ligand interaction^18^, the idea is that the MST signal is expected to increase with increasing protein concentration, until it reaches a maximum signal, corresponding to a 1:1 ligand to receptor ratio. Subsequently, a plateau should be observed. Since the concentration of D2R is unknown, we have chosen to express its protein concentration in terms of the total protein concentration present, determined by bicinchoninic acid assay (BCA). The protein concentration in this experiment ranged from 10 to 5 µg/mL.

**Figure 1:**
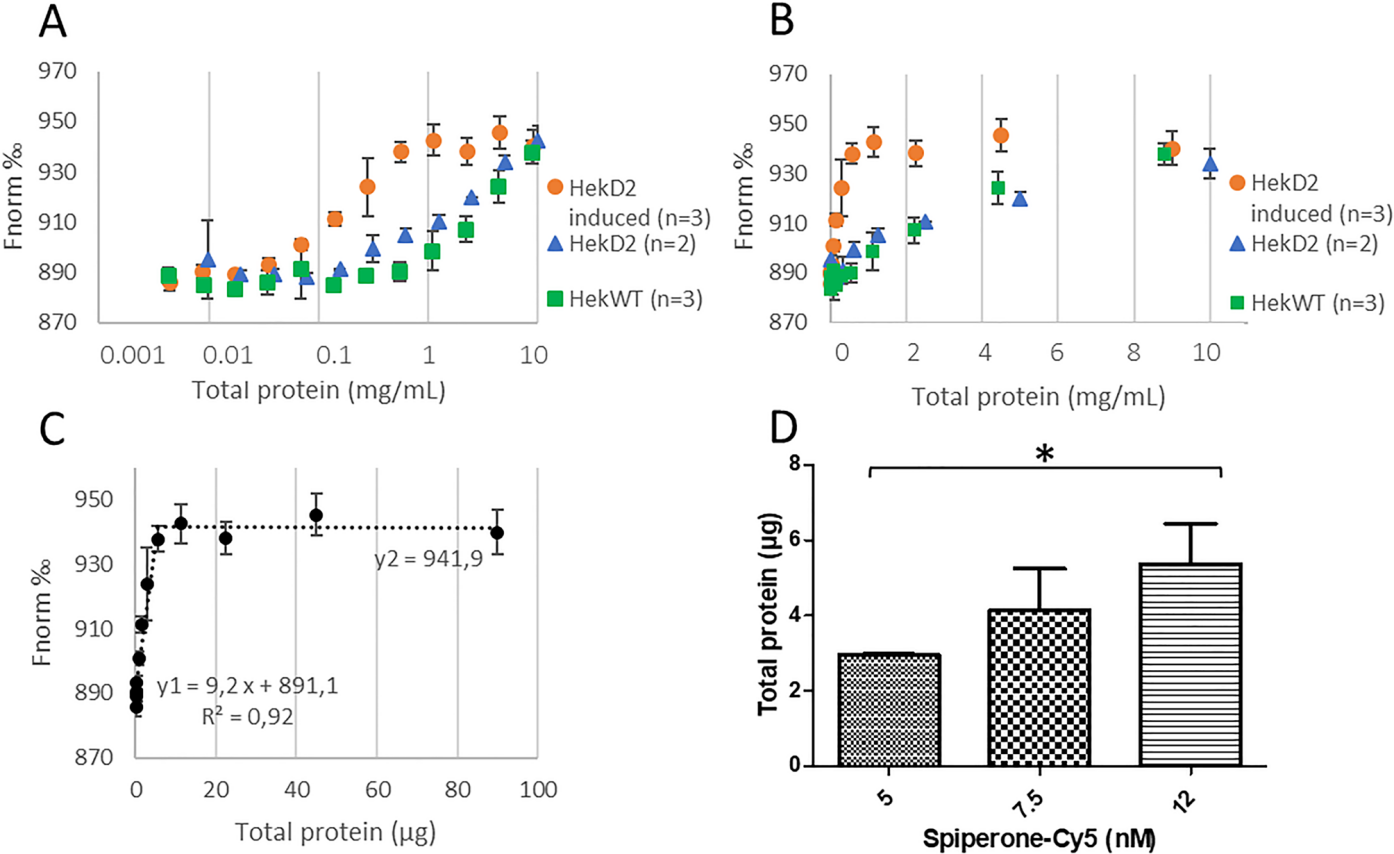
Determination of D2R concentration in cell membrane lysates using MST. Membrane fragments were serially diluted 1:1 and incubated for 1 h at room temperature with a fixed concentration of spiperone—Cy5 at 7.5 nM (**A**,**B**). Titration was performed with membrane fragments obtained from induced HekD2 (orange), incubated for 24 h with antibiotics (for details see materials and methods), non-induced HekD2 (blue) and HekWT (green) cells. Total protein concentration was measured by BCA assay, and reported in a logarithmic scale (**A**) or linear scale (**B**). The intersection point has been determined using the equations of the two curves obtained from 3 independent replicates, considering y1 = y2 (**C**). The value of x constitutes the total protein level corresponding to the spiperone—Cy5 concentration (7.5 nM in this example). The histogram (**D**) represents the mean intersection point for each spiperone-Cy5 concentrations ± standard deviation. A one-way analysis of variance has been used to compare the conditions. A significant difference is observed between 5 and 12 nM spiperone-Cy5 with P-value < 0,05 (*).

To ensure that the titration regime was reached for induced and not induced HekD2, three different spiperone—Cy5 concentrations were used for incubation with D2R membrane fragments (HekD2 diluted in the buffer): 5 (supplementary fig. S1 online), 7.5 (Fig. 1A,B), and 12 (supplementary fig. S1 online) nM. At very low membrane fragment concentrations, spiperone—Cy5 remained unbound. In the presence of D2R in the membrane fragments, a linear relationship between the protein concentration and the fluorescence signal is observed until the signal reaches a plateau. The intersection point corresponds to a condition where there is an equal concentration of D2R and spiperone—Cy5 (Fig. 1C). The concentration of D2R in the membrane fragments has been calculated using the intersection point of the mean curve obtained from 3 independent experiments (Fig. 1D). The calculated D2R concentration in HekD2 (antibiotics-induced) cell membrane fragments was 33.9, 37.9 and 38.8 pmol/mg of total protein with 5, 7.5 and 12 nM spiperone—Cy5, respectively. The results indicate, as expected, that D2R concentration is quite similar among the three measurements (Table 1), independent of the ligand concentration used. In parallel, similar experiment has been performed to determine the D2R concentration determined in non—induced HekD2 membrane fragments. Due to low D2R expression level, the difference between these samples and HekWT is small, in particular with 7.5 nM spiperone—Cy5. The D2R concentration has been estimated at 4 and 3 pmol/mg of total protein with 5 and 7.5 nM spiperone—Cy5, respectively. The D2R concentration in non-induced HekD2 membrane fragments was not determined at spiperone—Cy5 concentration of 12 nM as it was already difficult to saturate D2R with 7.5 nM spiperone—Cy5 in this sample. For the HekWT sample, spiperone—Cy5 binding to membrane fragments is observed for the highest total protein concentrations, however it does not reach a plateau (Fig. 1A,B).

**Table 1:** D2R concentration per total protein content.

[spiperone-Cy5] nM	[D2R] pmol/mg	
	Hek D2	Hek D2 induced
5	4	33.9
7.5	3	37.9
12	–	38.8

D2R concentration has been reported in relation to total protein content in membrane fragments, and calculated using the intersection point as previously described. Since titration regime was clearly obtained with induced HekD2, this concentration was obtained from three independent replicates for the three different spiperone—Cy5 concentrations (5, 7.5, 12 nM). On the other hand, the result for non-induced HekD2 may be considered as an estimation.

The D2R concentration of HekD2 induced was fixed at 37 pmol/mg (mean of all the measurements) for the second type of experiments to be performed by MST, aiming at determining the affinity of known D2R ligands to this receptor (dose response curves).

### Optimization of conditions for the measurement of dose response curves

**Figure 2:**
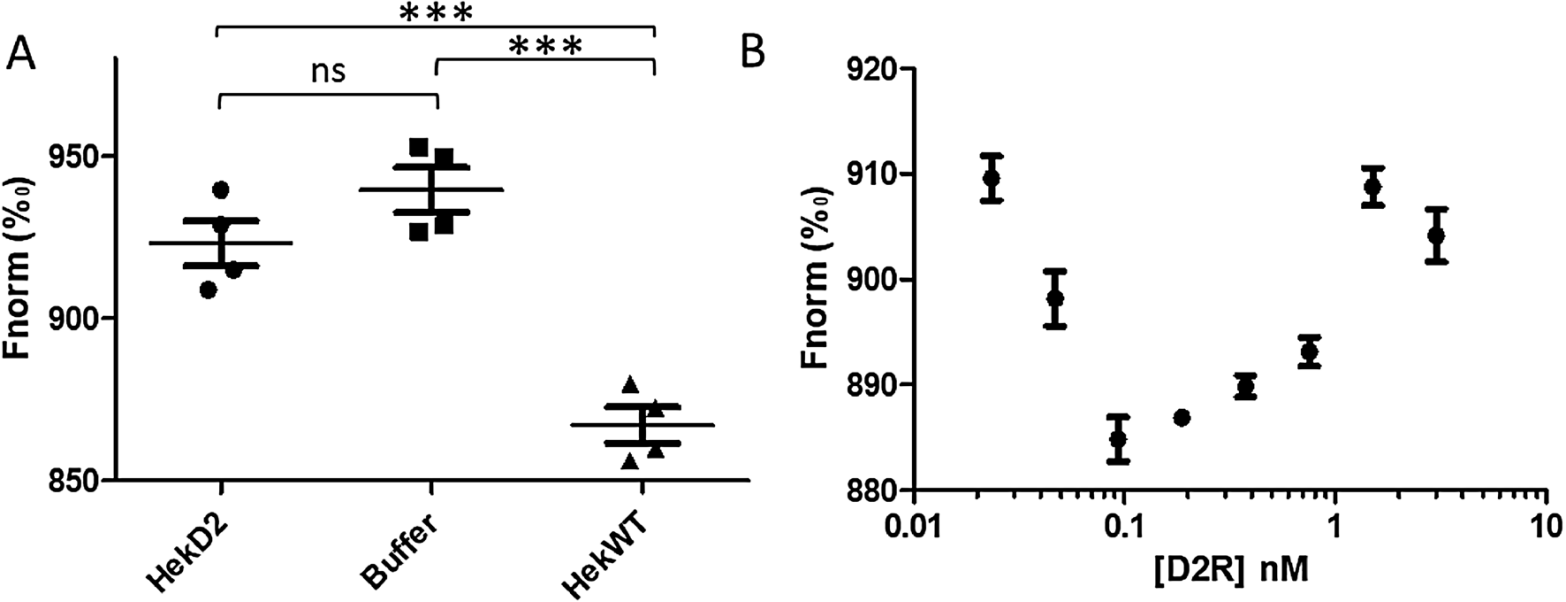
Binding check before saturation curve. The binding check allows the comparison of MST signal for 0.125 nM spiperone—Cy5 in presence of induced HekD2, buffer or HekWT (**A**). A one-way ANOVA analysis of variance has been used to compare the conditions. A significant difference is observed between HekWT and HekD2, and HekWT and buffer with P-value < 0.0001 (***). No significant difference is observed between HekD2 and Buffer conditions (ns). The total protein concentration was set similar in HekD2 and HekWT. Saturation curve obtained when HekD2 membrane fragments were diluted in buffer with 0.125 nM spiperone—Cy5 (**B**).

Binding checks were performed with different ligand concentrations in the absence (buffer only) or presence of membrane fragments expressing the D2R (HekD2) or not (HekWT), to optimize the amplitude of the measured MST signal. Ideally, the difference between the signal of the samples with and without D2R should be maximal, with a signal to noise ratio above 12. At a low spiperone—Cy5 concentration of 0.125 nM, the amplitude signal between HekD2 membrane fragments and the buffer is very small, as observed in Fig. 2A. However, when spiperone—Cy5 is diluted in HekWT membrane fragments, rather than in the buffer, a larger amplitude (about 5%) of the fluorescence signal between bound and not-bound spiperone is observed.

**Figure 3:**
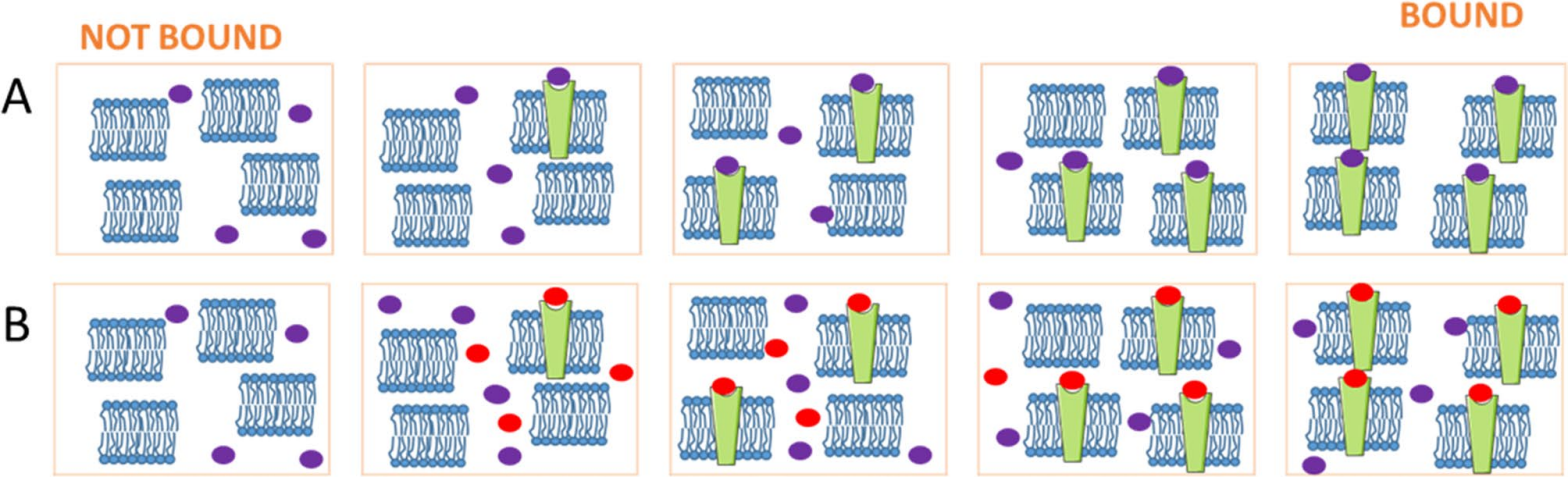
HekD2 membrane fragments (D2R in green) are diluted in HekWT membrane fragments (**A**). To characterize the presence or not of non-specific binding, a high concentration of non-labelled D2R antagonist, haloperidol (red), is added in each dilution point to compete with labelled ligand (violet) (**B**).

As a consequence, the dose response curve obtained by diluting directly the membrane fragments in the buffer is not optimal (Fig. 2B). The MST signal magnitude difference between the highest and the lowest concentration of D2R is very small (around 910 Fnorm), even if certain variations among them are observed. When dilution in the buffer is applied, not only the concentration of D2R varies, but also the concentration of the lipids and other proteins present in the membranes. Thus, the D2R induced cell membrane fragments were diluted with HekWT membrane fragments (with similar total protein concentrations) rather than the buffer, as illustrated in Fig. 3A.

### Determination of ligand binding affinity to D2R in cell membrane fragments by MST

**Figure 4:**
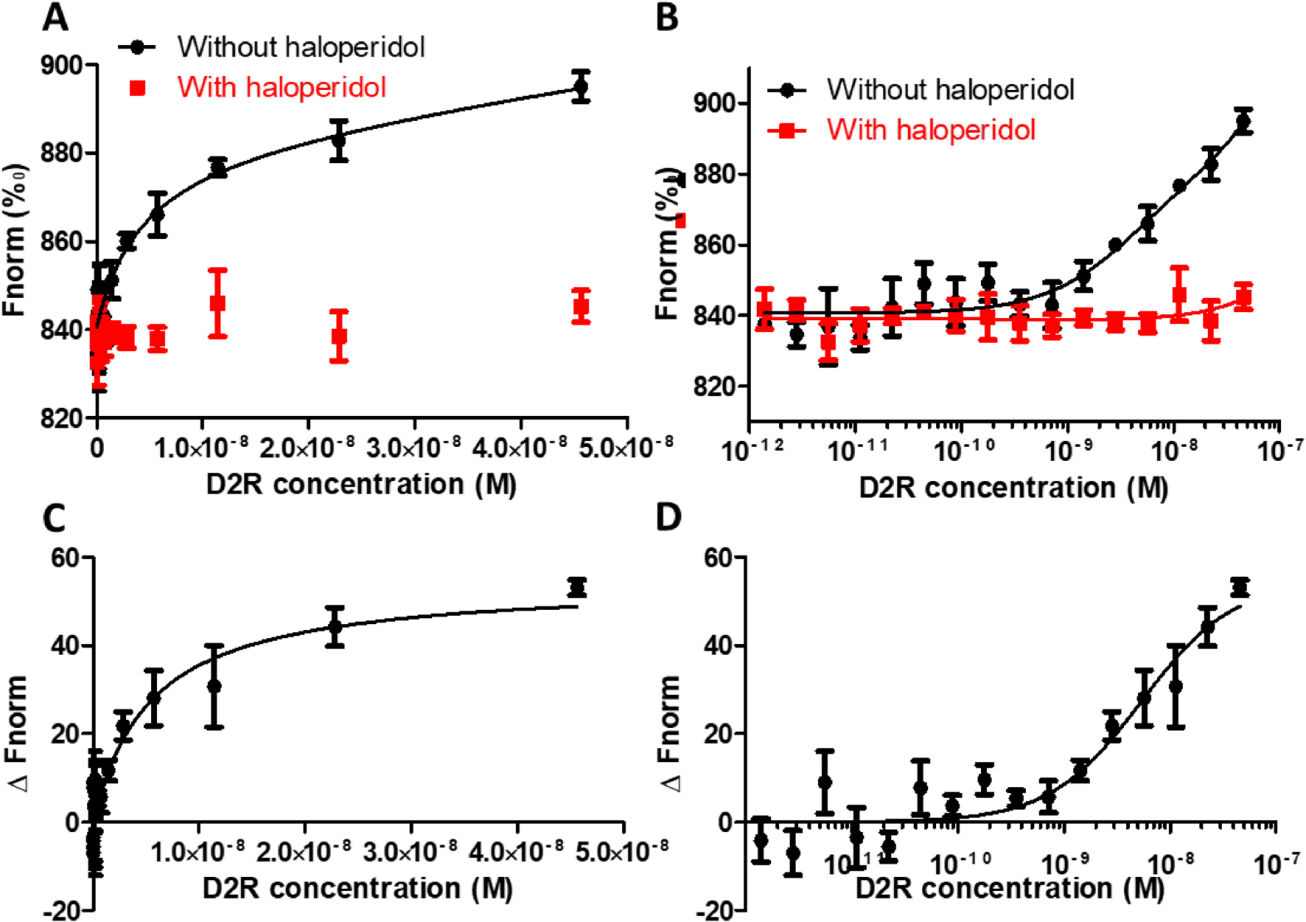
Dose response curves obtained using MST. Dose response curves were obtained with various D2R concentrations and a fixed concentration of 0.125 nM spiperone—Cy5, in presence (red) or absence (black) of 10 µM haloperidol (**A**,**B**, n = 4), and reported in a linear scale (**A**) or logarithmic scale (**B**). The normalized fluorescence (Fnorm) obtained in presence of haloperidol is subtracted from the signal obtained in absence of haloperidol for each individual experiment in order to obtain specific binding ΔFnorm (**C**,**D**, n = 4).

In this measurement, spiperone—Cy5 is maintained at a fixed concentration of 0.125 nM, which is well below the expected K_D_ value (6.6 nM, according to the supplier), while D2R concentration is varied from 10^–12^ to 10^–8^ M. Moreover, in order to discard any possibility regarding non-specific binding between the ligand and the cell membranes (as such ligands have strong affinity for lipids)^19^, experiments have been carried out both in the absence and in the presence of a D2R antagonist and a known antipsychotic, haloperidol (Fig. 3B). As shown in Fig. 4A,B, the MST signal rises with increasing D2R concentration in absence of haloperidol (black curves). On the contrary, in presence of haloperidol, the MST signal stays unchanged (red curves). The MST signal increase observed in the absence of haloperidol reflects the specific binding between spiperone—Cy5 and the D2R. By subtracting the non-specific binding from the total binding values (Fig. 4C,D), specific binding values can be obtained. The K_D_ determined by fitting the binding curve obtained from the mean MST signal without haloperidol (total binding) is 4.3 ± 1.3 nM, very close to 5.3 ± 1.7 nM, which has been obtained from the binding curve after subtracting the mean MST signal with haloperidol (non-specific binding) from the mean MST signal obtained without haloperidol (total binding).

## Discussion

Herein, we present a protocol to measure ligand binding affinity on integral membrane proteins, such as GPCRs, in conditions very close to their native environment, without any purification nor labelling procedure. The D2R has been used as a model GPCR, as it is a major therapeutic target, from antipsychotic to anti-emetic drugs.

MST has already been used to investigate GPCRs properties^9,15,16^. In general, the protein of interest is required to be solubilized, and specifically labelled following its purification. With non-purified protein of interest, the technique also requires specific labelling of the protein, for example, through a histidine tag. This is the strategy that was initially employed, to fluorescently label the histidine-tagged D2R to follow its interaction with non-labelled ligands. Preliminary experiments on membrane fragments obtained from *Pichia pastoris* yeast, overexpressing histidine-tagged RD2 specifically cyanine 5-labelled, in absence and presence of spiperone or quinpirole aiming at detecting their interaction by MST were unsuccessful. Indeed, it was not possible to detect a significant change in MST signal between bound and unbound states (supplementary fig. S4 online). We hypothesized that the reason why this failed is related with the fact that very small changes are expected upon binding of a small ligand to rather large and heterogeneous membrane fragments.

Still with the use of membrane fragments obtained from *Pichia pastoris* yeast expressing the D2R, we have decided to use the ligand as a tracer for the interaction, the fluorescent ligand spiperone -Cy5. In an attempt to reduce the size and heterogeneity of the D2R-containing membrane fragments, they were incubated with copolymer DiIsoButylene-Maleic Acid (DIBMA) to form lipid-polymer-protein particles (or nanodiscs). Unfortunately, strong non-specific binding of the fluorescent ligand spiperone -Cy5 was observed as a similar dose response was obtained following overnight incubation of the D2R-containing membrane fragments at 60 °C (in a way to denature the receptor) (Fig. S5A). Moreover, the dose response curves obtained with nanodiscs containing or not the D2R are almost the same (Fig. S5A, S5B1). This lead us to believe that non-specific binding was dominating the MST response. Therefore, binding of spiperone -Cy5 to liposomes (SUVs without copolymer or RD2) and to DIBMA copolymer alone was tested. The data show that only the polymer and not the lipid is responsible for the non-specific binding (Fig. S5B1, 2).

The next strategy that was adopted was to change the source of D2R, HEK cells overexpressing the D2R. D2R-containing membrane fragments in suspension, without any need for solubilisation, purification, labelling or further characterization procedures were thus employed. The use of cells with different receptor expression level allows to fine tune ideal D2R concentrations for MST measurements. Moreover, the use of empty HEK cells allows for negative control measurements. Using this strategy, determining D2R in the membranes was a prerequisite to measure a ligand binding affinity by MST. Measuring the membrane protein concentration in the sample using a fluorescent ligand requires the use of a ligand concentration higher than the K_D_, but lower than the concentration in the protein sample. These conditions lead to a titration regime, where the receptor is depleted in the solution due to its binding to the fluorescent ligand until there is no more free fluorescent ligand^20^. The intersection between the linear progression of Fnorm with increasing D2R concentration and the plateau, corresponds to a condition where there is an equal concentration of D2R and spiperone—Cy5, according to the stoichiometry of the reaction (Fig. 1C). We expect the stoichiometric regime for the complex D2R/spiperone to be 1:1 as one orthosteric binding site has been observed in the crystal structure determined for this complex^18^. Due to the value being close to the expected ligand K_D_, we were not sure to obtain a titration regime when using 5 nM final concentration of spiperone—Cy5, hence we repeated this experiment with higher ligand concentrations: 7.5 and 12 nM. The D2R concentration calculated from three different conditions (5, 7.5, and 12 nM of spiperone—Cy5) for induced HekD2 cell membranes was very much comparable (Table1), with a mean D2R concentration of 36.8 ± 2.6 pmol/mg. We were not able to precisely determine the D2R concentration, in non-induced HekD2 cell membrane, as D2R expression level was very low. A plateau has been reached for non-induced HekD2 incubated with 5 nM spiperone-Cy5 but this concentration is very close to the expected K_D_ value. It was not possible to increase membrane fragment concentrations in the samples to reach a saturation regime, as unspecific binding was observed for higher concentrations, as highlighted by the similarity between MST signals observed for HekWT (green) and HekD2 (blue) samples (Fig. 1A,B, SI1).

This may be considered as a limitation in our strategy, in which a certain expression level of the membrane protein of interest may be required. Depending of the system used in this strategy, the minimum expression level of the membrane protein is conditioned by the affinity of the fluorescent ligand to the protein, due to the titration regime being reached using concentrations higher than the K_D_ and lower than the maximal concentration of the protein in the sample. In our samples, a D2R expression level of about 37 pmol/mg of total protein allowed us to determine unknown concentrations, while non-induced HekD2 presenting approximately a 10 times lower expression level could not. Thus only induced cells were used in the following steps of our study. Contrary to the saturation regime required to determine the target concentration, an equilibrium regime was necessary to determine the K_D_. The concentration of the fluorescent ligand used for ligand binding affinity assay was ten times lower than the ones used for the titration of D2R. As previously observed, the thermophoresis phenomenon is linked to buffer conditions^12^, hence particular attention should be paid to avoid any potential variations between the different titration points. As an example, the MST response observed for spiperone-Cy5 in the absence (dilution in buffer only) and presence of HekD2 are the same (Fig. 2A). This is a consequence of the properties of the fluorescent tracer being very sensitive to their environment. It was therefore necessary in our study to dilute HekD2 fragments with the use of HekWT fragments, in order to maintain a constant concentration of total proteins and lipid membranes. In this configuration, only the concentration of D2R varies across the dilution, while the concentration of total protein (except the D2R) and lipid membrane remained stable. Using this experimental procedure, only the specific binding is being observed. To investigate if non-specific binding occurs, a high concentration of haloperidol, a non-labelled D2R antagonist, was added to the different dilution wells, in order to compete with spiperone for the receptor binding site. After subtraction of non-specific from total binding, the K_D_ value was determined to be 5.3 ± 1.7 nM, which is in very good agreement with other reported values (6.6 nM provided by the commercial supplier; 2.88 nM by Lane and coworkers)^21,22^.

This paper describes for the first time a protocol to determine the K_D_ between a ligand and a GPCR directly in membrane fragments, without any solubilisation, purification, or labelling step, but using MST only. This strategy may be implemented to determine the K_D_ of various membrane proteins, allowing an alternative strategy to the panel already available.

## Methods

### Materials

Fetal bovine serum (FBS), trypsin/EDTA (0.05%/0.02%), ethylene diamine tri acetic acid (EDTA), Dulbecco’s Phosphate Buffered Saline (DPBS), Protease inhibitors cocktail (PI), Tween 20, Tetracyclin, Penicillin–Streptomycin (Pen-Strep) were purchased from Sigma-Aldrich (Munich, Germany). Blasticidin and Hygromycin B were obtained from Invivogen. Tag-Lite® dopamine D2 receptor red antagonist (spiperone—Cy5) was purchased from Cisbio Bioassays. The total protein concentration has been determined using the Pierce™ BCA protein assay kit provided by ThermoFischer Scientific.

### Mammalian cell culture

Hek293T cells (HekWT) and the stably transfected D2R-expressing Hek-293 T (HekD2) cells, were kindly provided by Prof. Jonathan A. Javitch (Department of Pharmacology, Columbia University, New York, USA). Both cell lines were maintained in DMEM supplemented with 10% FBS, 1% Pen-Strep, in a water-saturated atmosphere (37 °C, 95% air, 5% CO2). To enhance D2R expression level from HekD2 cell line (induced HekD2), cells were incubated for 24 h with the following antibiotic cocktail: hygromycin 0.1 mg/mL, tetracyclin 0.01 mg/mL, blasticidin 0.015 mg/mL.

### Preparation of membrane fragments

When cells were 80% confluent in T175 cell culture flasks, they were dissociated with 1.5 mL DPBS containing 1 mM EDTA. Cells were collected by centrifugation and pellets were incubated in PBS 20 mM, EDTA 2 mM, PI, for 30 min on ice. Samples were homogenized through 100 up and down movements using Potter homogenizer on ice and centrifuged 10 min 400×*g* at 4 °C. Pellets were collected and a second time homogenized with Potter on ice and centrifuged 10 min 400×*g* at 4 °C. Supernatants were centrifuged 45 min at 20 000×*g* at 4 °C to pellet membrane fragments. The pellets were then collected in a smaller volume with PBS, tween 0,05%, PI in order to obtain a total protein concentration of ~ 10 mg/mL, as controlled by BCA for each sample. Samples were stored at − 80 °C before use.

### Microscale thermophoresis

MST experiments were performed on a Monolith NT. 115 (NanoTemper Technologies GmbH) using a red filter set. All dilutions were prepared to ensure that no other gradient (salt, glycerol, DMSO, etc.) was created during buffer mixing. To minimize adsorption of the sample to material, low retention tips and tubes were used, and Tween 20 at 0.05% was added to the PBS, buffer used to dilute all components. After mixing the different components, the samples were incubated at room temperature for 30 to 60 min before loading into standard capillaries (Nanotemper Technologies). For binding check, HekD2 membrane fragments, buffer, or HekWT, adjusted to HekD2 regarding total protein concentration, was mixed with an equal volume of spiperone—Cy5 to obtain a final concentration of 0.125, 5, 7.5, 12 nM. After an incubation time of 1 h, capillaries were loaded and the LED was set to 20% for 0.125 nM, and 1% for 5, 7.5 and 12 nM, using medium MST power. For the receptor titration assay, the protein sample at various concentrations ranging from ~ 10 mg/mL total protein to ~ 5 µg/mL was mixed with a fixed concentration of the fluorescent ligand. The fluorescent ligand, spiperone—Cy5, was added at a final concentrations of 5, 7.5, or 12 nM to each dilution point. An incubation time of 1 h was taken before capillary loading. The LED power was set to 1% and MST power to “medium”. The intersection point has been determined using Excel, from the curves obtained from the mean of three replicates, as shown in Fig. 2B. In binding affinity assays, samples obtained from HekD2 were serially diluted 1:1 (v:v) in HekWT and adjusted at the same total protein concentration, before adding spiperone—Cy5 at a final concentration of 0.125 nM, with or without haloperidol 10 µM (for competition assays). After a 2 h incubation time, capillaries were loaded. The LED power was set to 20% and MST power to “medium”. The K_D_ has been determined using the one site—specific binding equation from GraphPad software, from the curves obtained from the mean of three replicates. In all MST protocols, MST-on time is measured at 1.5 s after infrared laser heating.

## Supplementary Information

Supplementary Information.
