# Supplementary material for: An original approach to measure ligand/receptor binding affinity in non-purified samples
Source: Sci Rep. 2022 Mar 30;12:5400. doi: 10.1038/s41598-022-09217-6 (PMC8967833; doi:10.1038/s41598-022-09217-6)
Supplement: Supplementary file 1 — Supplementary Information. [file 41598_2022_9217_MOESM1_ESM.docx]

*Supplementary information*

**An original approach to measure ligand/receptor binding affinity in non-purified samples**

**Estelle Rascol**^1^***, Anouk Dufourquet**^1^**, Rim Baccouch**^1^**, Pierre Soule**^2^**, Isabel D. Alves**^1^

1 Univ. Bordeaux, CNRS, Bordeaux INP, CBMN, UMR 5248, F-33600 Pessac, France

2 Application Team, NanoTemper Technologies GmbH, Munich, Germany

*estelle.rascol@u-bordeaux.fr


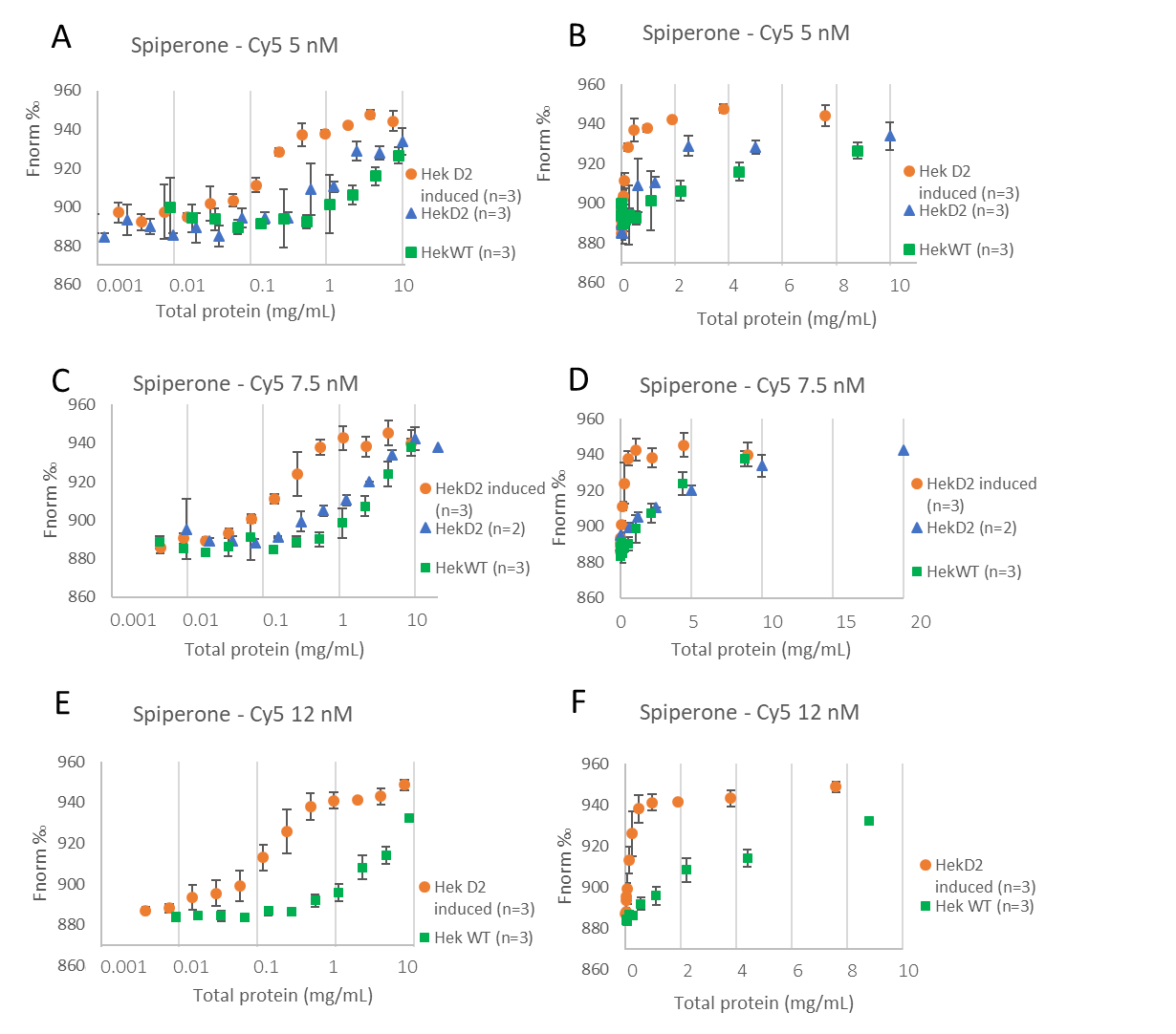


Figure S 1: Determination of the D2R concentration in cell membrane lysates using MST. Membrane fragments were serially diluted 1 : 1 and incubated for 1h at room temperature with a fixed concentration of spiperone - Cy5 at 5 (A, B), 7.5 (C, D), and 12 (D, E) nM. Titration was performed with membrane fragments obtained from induced HekD2 (orange), incubated 24h with antibiotics (for details see materials and methods), non-induced HekD2 (blue) and HekWT (green) cells. Total protein concentration was measured by BCA assay, and reported in a logarithmic scale (A, C, E) or linear scale (B, D, F).


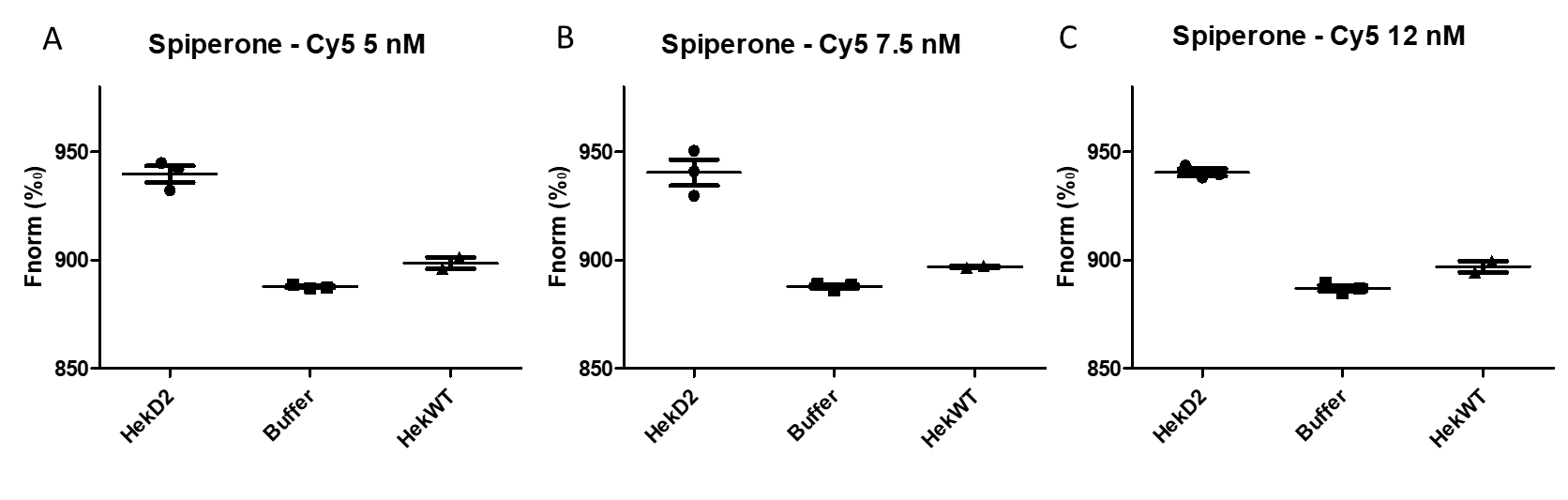


Figure S 2: Binding checks performed to optimize amplitude signal of MST traces. The binding check allows the comparison of MST signal for different spiperone - Cy5 concentrations in presence of induced HekD2, buffer or HekWT. The total protein concentration was set similar in HekD2 and HekWT.


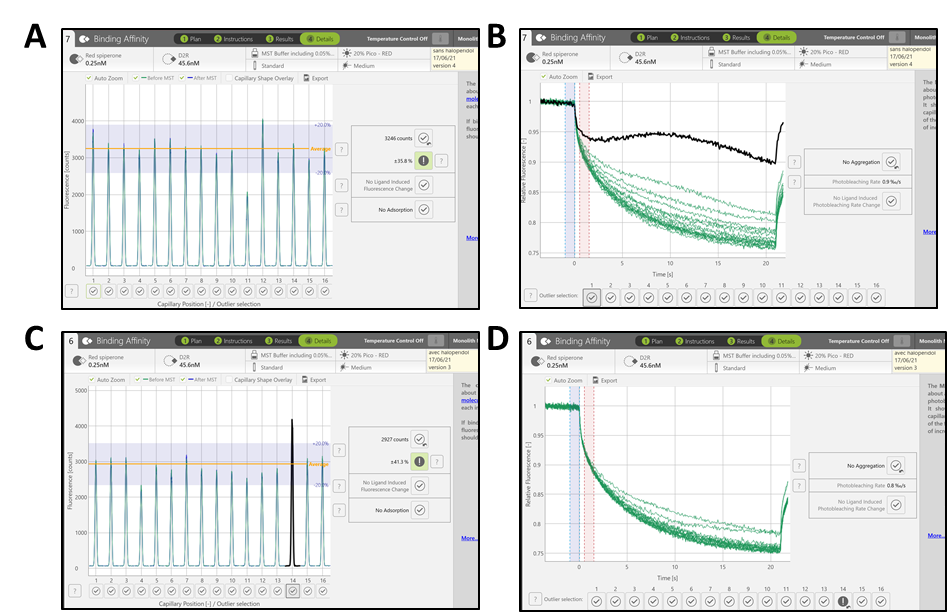


Figure S 3: Example of capillary scans (A, C) and MST traces (B, D) obtained for dose-response curves in absence (A, B) or presence (C, D) of 10 µM haloperidol. When MST traces resembles the first capillary in absence of haloperidol, on panel B, the trace was not taken into account. When fluorescence was higher or lower than 20% of the average, the capillary was not taken into account. 14^th^ capillary in presence of haloperidol on panel C has been removed for analysis. The data obtained from 4 independent experiments allow the formation of a complete dose-response curve presented on figure 4.

In parallel to the experiments reported in this paper, performed on Hek293 mammalian cells expressing or not the D2R, some experiments reported here were with membrane fragments obtained from *Pichia pastoris* yeast overexpressing the D2R.

Histidine tagged D2R, expressed in Pichia pastoris yeast, has been labelled directly on membrane fragments. Crude yeast membranes were incubated for 30 min in the dark at room temperature with 100 nM red NTA labeling kit, provided by Nanotemper. After that, the membranes were incubated with 5 µM final concentration of quinpirole or spiperone (two different D2R ligands). As observed from binding checks comparing the fluorescence signal of *Pichia pastoris* membrane fragments containing the labeled D2R in the absence (buffer) and presence of quinpirole (SI 3-A) or spiperone (Si 3-B), no significant difference has been observed.


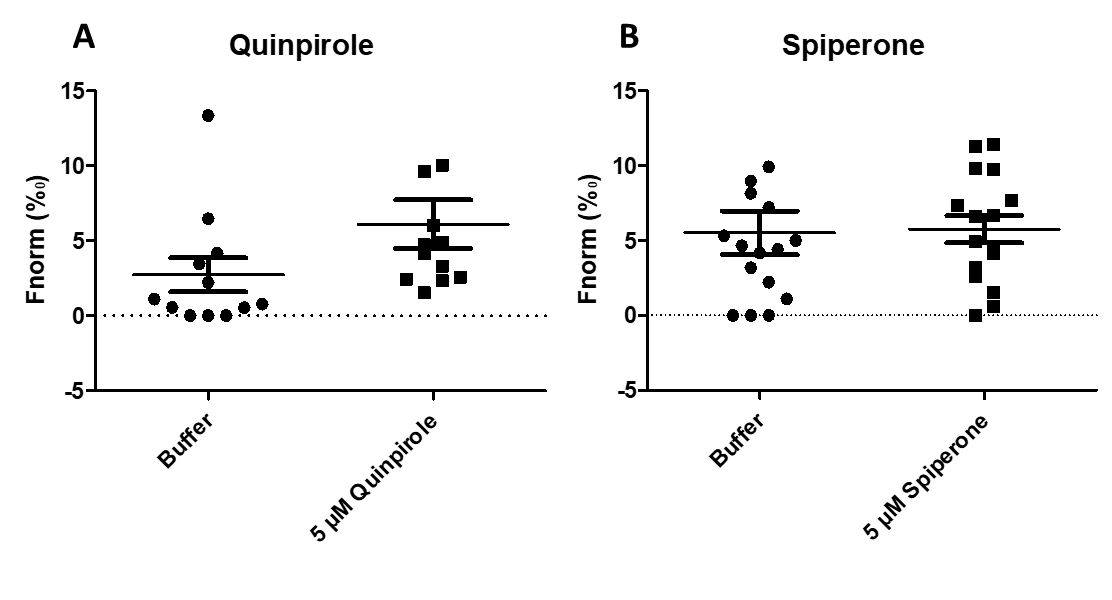


Figure S 4: Binding checks comparing the fluorescence signal of *Pichia pastoris* membrane fragments containing the labelled D2R in absence (buffer) and presence of quinpirole (A) or spiperone (B). No significant difference is observed.

D2R, expressed in *Pichia pastoris* yeast has been solubilized directly from crude membranes using DiIsoButylene-Maleic Acid (DIBMA) copolymer. The membranes were diluted in buffer containing DIBMA at a final concentration of 3.5% (m/v) and incubated under agitation at 4°C for 12 h. Next, membrane copolymer nanodiscs were serially diluted and put in contact with 2 nM spiperone-Cy5 to obtain a binding curve (SI 4-A1). After >12h at 60°C, the saturation remained identical indicating that the observed curve was not specific to the presence of functional D2R, but may correspond to non-specific binding. Non-specific binding has been characterized by doing the same experiment without D2R (SI2-B). A binding curve was observed for nanodiscs obtained by incubating the DIBMA copolymer directly with multilayer vesicles (MLVs) formed with Egg Phosphatidylcholine (SI 4-B1), that corroborate the non-specific hypothesis. Other control experiments were performed with Egg Phosphatidylcholine liposomes alone (small unilamellar vesicles, SUV), or with DIBMA copolymer alone. The fluorescent ligand seems to bind non-specifically to DIBMA (SI 4-B3) and not to the lipids (SI 4-B2).


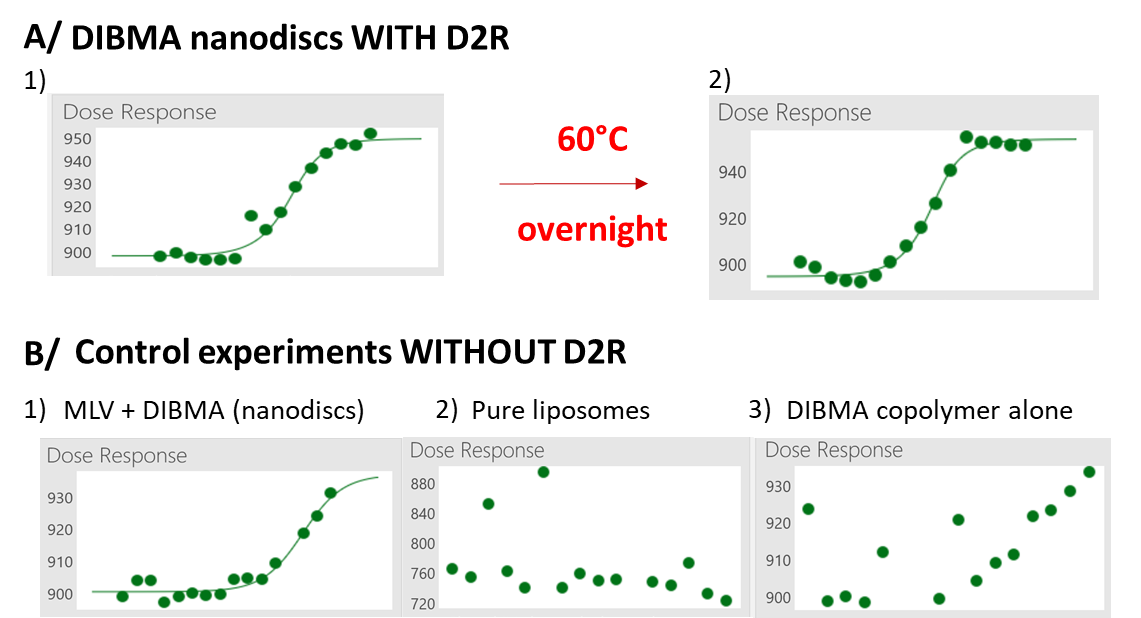


Figure S 5: Binding curves of serially diluted membrane systems containing the D2R (A) or not (B), following incubation with 2 nM spiperone-Cy5: membrane copolymer nanodiscs containing the D2R, before (A1) or after (A2) incubation overnight at 60°C, control experiments without D2R (B): MLV incubated with DIBMA (nanodiscs without D2R) (B1), liposomes only (SUV) (B2) or DIBMA copolymer alone (B3). The fluorescent ligand seems to bind non-specifically to DIBMA (B3) and not to the lipids (B2).
